# In Search of a Lost Father: Adrien Proust (1834–1903), An Almost Forgotten Public Health Pioneer

**DOI:** 10.3390/vaccines10050644

**Published:** 2022-04-20

**Authors:** Donatella Lippi, Elena Varotto, Sara Boccalini, Angela Bechini, Francesco Maria Galassi

**Affiliations:** 1 Department of Experimental and Clinical Medicine, School of Sciences of Human Health, University of Florence, 50134 Florence, Italy; donatella.lippi@unifi.it; 2 Archaeology, College of Humanities, Arts and Social Sciences, Flinders University, Adelaide 5001, Australia; elena.varotto@flinders.edu.au (E.V.); francescom.galassi@flinders.edu.au (F.M.G.); 3 Department of Health Sciences, University of Florence, Viale GB Morgagni 48, 50134 Florence, Italy; sara.boccalini@unifi.it

**Keywords:** history of medicine, hygiene, Proust, public health, tradition, pandemic emergency

## Abstract

Objectives: In this communication, we wish to remember the important historical role played by Marcel Proust’s father, the now mostly forgotten Achille-Adrien Proust (1834–1903). Study Design and Methods: His career, scientific interests and, above all, his brilliant intuitions and suggestions in the fight against cholera in the 19th century are recalled. Results and Conclusions: His role in the promotion of a globally effective vision of public hygiene and health is stressed as a bright example for modern physicians fighting contemporary epidemics.

## 1. Introduction

In the centenary of the death of the celebrated French writer Marcel Proust (1871–1922), the author of the masterpiece “À la recherche du temps perdu” (“In Search of Lost Time”, 1913–1927), it is important to remember his father, the famous physician and public health official Achille-Adrien Proust (1834–1903), who was a pioneer of public hygiene, setting up a fruitful policy against the spread of epidemics. The importance of this learned author has been overshadowed by his son’s posthumous fame [1]. His insights in the means of containing the contagion offer interesting points of comparison with the current COVID-19-related situation.

## 2. His Life

**Figure 1:**
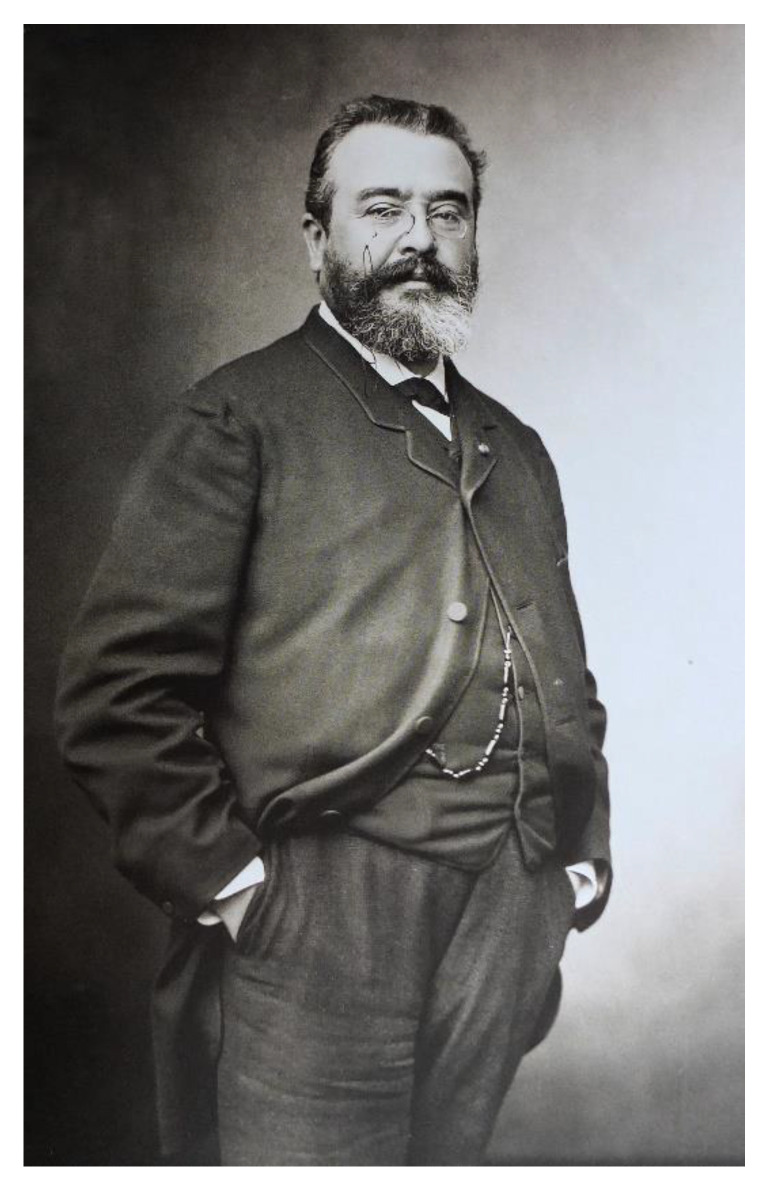
Photograph showing Adrien Achille Proust (1834–1903) taken on 26th November 1886. Image in the public domain from Wikipedia. Available online at: https://en.wikipedia.org/wiki/Adrien_Proust#/media/File:Adrien_Proust_1834–1903.jpg (last accessed on 27 February 2022).

**Figure 2:**
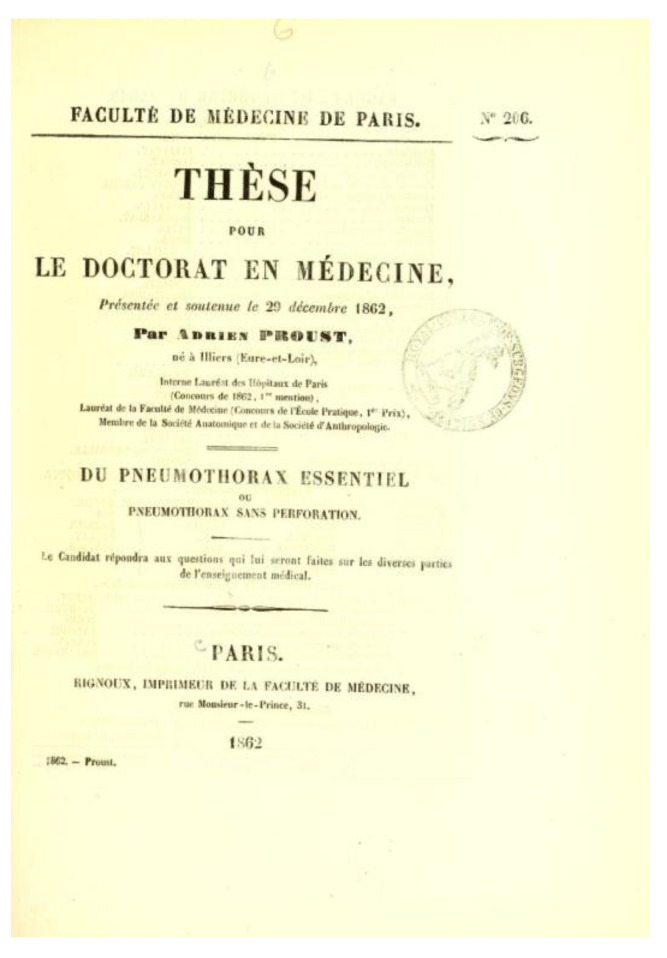
Frontispiece of Proust’s 1862 medical doctoral dissertation *Du pneumothorax essentiel ou pneumothorax sans perforation*. The text is freely accessible on the Wellcome Collection’s website: https://wellcomecollection.org/works/nybrbuus (last accessed on 27 February 2022).

Achille-Adrien Proust studied medicine in Paris, where, in 1862, he obtained his medical doctorate, discussing a thesis about “Idiopathic Pneumothorax” (Figure 1 and Figure 2) [2]. He was appointed Chief of Clinic in 1863, *professeur agrégé* in 1866, physician at the Central Office in 1867. In 1869 (Fourth cholera pandemic, 1863–1875), the Minister of Agriculture and Commerce sent him on a mission to Russia and Persia to study the means of fighting against the invasion of cholera. In France, cholera had reached epidemic proportions during the period 1830–1845 (Second cholera pandemic, 1829–1837) and during the siege of Paris in 1870. A pupil of the physician Pierre Fauvel (1830–1895), who was the first to promote the idea of the arrival of cholera in Europe from India, Proust turned early to hygiene, and thus prepared himself for a long time for the studies proper to the chair that he was to occupy later and to the great scientific missions that were to be entrusted to him. During this journey, he also visited Athens, Constantinople, Messina and several locations in Germany.

When he came back, he was awarded the Legion of Honour from the Empress-Regent Eugénié [3]. That the same year, he married Jeanne-Cléméncé Weil, who bore him two sons: Marcel and Robert, the latter of whom would become a distinguished urologist and gynaecologist. In 1879, Adrien Proust was elected to membership of the Academy of Medicine and became General Inspector of Sanitary Services in 1884. In 1885, he received his full professorship of Hygiene at the Medical Faculty of the University of Paris. In this capacity, he attended, as a delegate of the French government, the many international health conferences which were held from 1874 to 1903.

At a conference held in Venice in 1892 he played a considerable role by adopting, against certain proposals of England, conclusions which indicated, with precision, the method to be followed to reconcile the interests of public health and universal commerce, that often conflicted.

Endowed with great activity, Proust published numerous works, both on pathology and on hygiene.

Among his medical works, his doctoral thesis on essential pneumothorax [4], his thesis for the public competition to become *professeur agrégé* on the different forms of softening of the brain [5] and a large number of accounts regarding neurological problems can be cited.

Nonetheless, international hygiene was Dr Proust’s great concern, and since his trip to the East, he never ceased to occupy himself with the means of defence that should be opposed to major epidemics, as evidenced by the brilliant reports that he often communicated on the origin and progress of these scourges through different peoples. His numerous publications on this subject are summarized in an important treatise on private and public hygiene, in which ethnography, demography and international hygiene, which has taken on such great development in recent years, are united [6]. Written with clarity and simplicity, this book was a great success.

In his various works about international hygiene, Proust endeavoured to illustrate the terrestrial routes and the sea routes followed by the great epidemics. He pursued the creation of an international health union, which, while respecting the sovereignty of States and their sensitivities, would have a non-executive but moral power, capable of overcoming resistance and coordinating the efforts of the various governments.

He died in Paris on 26 November 1903.

## 3. Hygiene in France

At the middle of the 19th century, France was the leader of Western clinical medicine and played a very important role in hygiene, too [7]. As in many other European countries, the development of hygiene and epidemiology turned into the foundation of public health services, and in France, it resulted in the first Law on Public Health in 1902. Two facts must be underlined: In 1870, France was defeated in the Franco-Prussian war.

In this conflict, more deaths of French soldiers occurred due to the smallpox epidemic than to the war because the French military contingent had not been vaccinated, unlike the German one. From this period onwards, doctors engaged in politics in large numbers as deputies and senators.

In 1877, the *Société de médecine publique et d’hygiène professionnelle* was founded in Paris. Apollinaire Bouchardat, professor of Hygiene at the Sorbonne Faculty of Medicine, joined it, together with the greatest exponents of French medicine and biology and some members of the *Académie de médecine*, the *École vétèrinaire*, the *École d’Anthropologie,* the *Conservatoire des arts et métiers*, as well as the head of the *Statistique municipale de la Ville de Paris* service and numerous politicians.

In 1889, in fact, the *Societé* was divided into different sections, each of which dealt with a specific subject: *hygiène de l’enfance, hygiène urbaine et rurale, hygiène industrielle et professionnelle, prophylaxie des maladies contagieuses, hygiène alimentaire, hygiene internationale et administrative and démographie et statistique*.

This public health process was possible thanks to a widespread hygienist movement supported by the works of Pasteur (1822–1895), which paved the way to the birth of microbiology and provided the explanation of the role played by microorganisms in the emergence and development of disease in humans and animals.

The concept of “hygiene”, between the end of the 19th century and the beginning of the 20th century did not only indicate the set of individual and public prevention rules against diseases but had a broader value, including a real “lifestyle”.

From this point of view, the *Bibliothèque d’Hygiène Thérapeutique*, published by Masson in Paris, which included many texts dedicated to various pathologies, from gout to tuberculosis, from syphilis to obesity, can be considered paradigmatic. The cover of the books included in the series was illustrated with a representation of Hygieia (Ὑγεία), the Greek goddess of health. The purpose of this collection was to form a true treatise on “Therapeutic Hygiene”, each volume being dedicated to a single disease or to a single group of diseases. Adrien Proust was the editor.

## 4. Adrien Proust as a Hygienist

In 1869, the minister of Agriculture and Commerce sent Adrien on a hard journey to Russia and Persia to determine by which route cholera had arrived from Russia during the 1832 epidemic. He returned to Paris via Mecca, Turkey, and Egypt, thus following the route of the epidemics of 1849 and 1866.

He traced the routes of the great epidemics, taking an interest in their spread during the pilgrimage to Mecca, and paying great attention to hygiene in transport, especially maritime transport.

Proust proposed an international endeavour to prevent the spreading of epidemics, reactivating the sanitary cords, theorizing systematic containment and rigorous sequestration, interrupting communications by land or by sea. In Proust’s view the international hygiene was a much bigger question than the nations’ frontiers, and he considered countries where the cholera epidemic began spreading as a sort of barrier to protect Europe, especially when the first cases would have been identified and early interventions of isolation and other preventive measures would have been adopted [1].

As he had studied the outbreak phenomena well, he maintained that quarantines “close to the starting point of the disease” are of unquestionable effectiveness but if prescribed too late, they can even become harmful. He recommended keeping distance from the sick people and the washing hands and face frequently. The “cordon sanitaire” (sanitary cordon) would create a border whereby nations would collaborate to enforce a strict quarantine for ships entering their waters. However, the Suez Canal Company raised strong objections to his proposals, and so a fifth cholera epidemic occurred in 1884, carried by ships that had passed through the Canal. The spreading was limited to Toulon and Marseilles, as State-enforced cordons sanitaires around these towns successfully prevented the spread of cholera to other parts of France.

As Inspector General of Sanitary Services, Adrian Proust participated in the Conference of Vienne (1874), proposing the establishment of an international health organization permanent office known as the Office International d’Hygiene Publique.

During the ninth conference, held in Paris in 1894, Proust gave a summary of the outcome of previous international sanitary conferences since 1851, and insisted on the need not only for sanitary surveillance of the Red Sea and the Persian Gulf but also for adequate precautions to be applied to pilgrim ships at their ports of departure. However, too many European countries had a direct interest in the Mecca Pilgrimage: Britain, the Netherlands, France and Russia and Austria-Hungary. During the conference, Robert Koch (1943–1910) affirmed that the 10-year struggle over the nature of cholera was at an end, but different opinions persisted for a long time among “contagionists” and “localists” [8].

Despite Proust’s efforts, at the tenth Venice conference in 1897, his idea was not met with success, and the proposals made for cholera containment were only arranged for plague: It was only in 1908, 5 years after Proust’s death, that a specific convention was signed, providing a coordinating body to fight against the terrible triad of plague, yellow fever and cholera.

## 5. Conclusions

Nowadays, we have vaccines available, but in the past, quarantine and sanitation were the only methods to limit the spread of epidemics. We have learned from the epidemics of the past and from the COVID-19 global emergency that, to stop pandemics, we must control the circulation of host and virus: Inter-human contagion and cellular attack depend on the transfer of people across borders and viruses across cell membranes [9]. In the case of epidemic outbreaks, the speed of information and decision making is rewarding. Currently, we can rely on the International Health Regulations (IHRs), first adopted by the World Health Assembly (WHA) in 1969 and last revised in 2005, (following the 2002–2003 SARS outbreak), during the 58th WHA [10]. The IHRs have the scope “to prevent, protect against, control and provide a public health response to the international spread of disease in ways that are commensurate with and restricted to public health risks, and which avoid unnecessary interference with international traffic and trade”. As a matter of fact, the IHR Emergency Committee for COVID-19 held its first meeting on 22 and 23 January 2020, and following the second meeting, on 30 January 2020, the WHO advised States to “detect the disease early, isolate and treat patients, trace contacts and reduce social contacts” [11]. Despite the WHO’s rapid global alert, the COVID-19 experience showed how the international community needs to guarantee that countries are better prepared for preventing, monitoring, responding to and recovering from any future public health emergencies [12]. It is not for nothing that Adrien Proust, in his role of public health pioneer, is mentioned in “Love in the Time of Cholera”, the 1985 novel by Gabriel García Márquez, as the “most outstanding epidemiologist of his time”. Rediscovering his lessons, like those of other innovators/forerunners of the past [13,14] and, more generally, the historical presentation of infectious diseases [15], may be helpful for physicians fighting new infectious enemies in the 21st century.
